# Silver Nanoparticles Conjugated with Contact Lens Solutions May Reduce the Risk of *Acanthamoeba* Keratitis

**DOI:** 10.3390/pathogens10050583

**Published:** 2021-05-11

**Authors:** Edyta B. Hendiger, Marcin Padzik, Inés Sifaoui, María Reyes-Batlle, Atteneri López-Arencibia, Diana Zyskowska, Marta Grodzik, Anna Pietruczuk-Padzik, Jacek Hendiger, Gabriela Olędzka, Lidia Chomicz, José E. Piñero, Jacob Lorenzo-Morales

**Affiliations:** 1 Department of Medical Biology, Medical University of Warsaw, Litewska 14/16, 00-575 Warsaw, Poland; edyta.hendiger@wum.edu.pl (E.B.H.); d-zyskowska@wp.pl (D.Z.); gabriela.oledzka@wum.edu.pl (G.O.); lidia.chomicz@wum.edu.pl (L.C.); 2 Instituto Universitario de Enfermedades Tropicales y Salud Pública de Canarias, Departamento de Obstetricia, Ginecología, Pediatría, Medicina Preventiva y Salud Pública, Toxicología, Medicina Legal y Forense y Parasitología, Universidad de La Laguna, Av. Astrofísico Francisco Sánchez S/N, 38203 Tenerife, Spain; isifaoui@ull.edu.es (I.S.); mreyesbatlle@gmail.com (M.R.-B.); atlopez@ull.edu.es (A.L.-A.); jpinero@ull.edu.es (J.E.P.); jmlorenz@ull.edu.es (J.L.-M.); 3 Department of Nanobiotechnology and Experimental Ecology, Institute of Biology, Warsaw University of Life Sciences, 02-787 Warsaw, Poland; marta_grodzik@sggw.pl; 4 Centre for Preclinical Research and Technology (CePT), Department of Pharmaceutical Microbiology, Faculty of Pharmacy, Medical University of Warsaw, Banacha 1B, 02-097 Warsaw, Poland; anna.pietruczuk-padzik@wum.edu.pl; 5 Faculty of Building Services, Hydro, and Environmental Engineering, Warsaw University of Technology, 20 Nowowiejska Street, 00-653 Warsaw, Poland; jacek.hendiger@pw.edu.pl

**Keywords:** contact lenses, contact lens solutions, silver nanoparticles, *Acanthamoeba* keratitis

## Abstract

*Acanthamoeba* keratitis (AK), a severe sight-threatening corneal infection, has become a significant medical problem, especially among contact lens wearers. The disease manifests as eye pain, congestion, blurred vision, lachrymation, and ring-shaped infiltrates of the cornea, and can lead to permanent blindness. Inappropriate habits of contact lens users may result in an increased risk of AK infection. The anti-amoebic efficiency of popular multipurpose contact lens solutions is insufficient to reduce this risk. An effective and non-toxic therapy against AK has not yet been developed. The prevention of AK is crucial to reduce the number of AK infections. Nanoparticles are known to be active agents against bacteria, viruses, and fungi and were also recently tested against protozoa, including *Acanthamoeba* spp. In our previous studies, we proved the anti-amoebic and anti-adhesive activity of silver nanoparticles against *Acanthamoeba castellanii*. The aim of this study is to evaluate the activity, cytotoxicity, and anti-adhesive properties of silver nanoparticles conjugated with five commonly used multipurpose contact lens solutions against the *Acanthamoeba castellanii* NEFF strain. The obtained results show a significant increase in anti-amoebic activity, without increasing the overall cytotoxicity, of Solo Care Aqua and Opti Free conjugated with nanoparticles. The adhesion of *Acanthamoeba* trophozoites to the contact lens surface is also significantly reduced. We conclude that low concentrations of silver nanoparticles can be used as an ingredient in contact lens solutions to decrease the risk of *Acanthamoeba* keratitis infection.

## 1. Introduction

*Acanthamoeba* spp. can invade the human eye and cause a dangerous corneal infection, named *Acanthamoeba* keratitis (AK) [1,2,3]. The amoebae are commonly detected in water, including swimming pools and air conditioners, as well as in soil and dust. More than 90% of AK cases are diagnosed in the contact lens user population [1,2,3]. The AK infection rate has been increasing over years from 17 to 70 per million among contact lens wearers worldwide [2]. Poor education on proper contact lens management leads to inappropriate user habits, such as swimming with contact lenses, wearing them over the recommended time limit, or cleaning them in tap water. Contamination of the contact lenses with amoebae during storage or while wearing may be the first step in AK development [2,3,4]. Popular contact lens solutions mainly include topical anti-fungal and anti-microbial ingredients and are not fully efficient against *Acanthamoeba* trophozoites and cysts. Improvements in these formulations are urgently needed to limit AK infections [5].

Wearing contact lenses may cause micro-corneal damage and ulcerations, which act as anchor points for *Acanthamoeba* adhesion. The amoebae can simply transfer from the contact lens surface to corneal epithelial cells. AK infection affects corneal stroma and finally leads to nerve infiltration [6,7]. The advanced stage of AK manifests itself as eye pain, lacrimation, redness, eye congestion, blurred vision, and a foreign-body sensation. Ultimately, *Acanthamoeba* causes massive ocular injury and can result in permanent vision loss. The only pathognomonic symptom is the ring-shaped infiltrate that occurs in only 50% of AK cases [4]. The lack of other specific symptoms fosters misdiagnoses and delays recovery time, thus prolongs treatment duration [3,8,9]. 

Anti-AK drugs recommended by the CDC involve diamides and biguanides. The most popular are polyheksametylene biguanide (PHMB) and chlorhexidine. In the early stage of AK, a combination of chlorhexidine with dibromopropamadine, hexamidine, propamidine isethionate, and neomycin shows the most promising results [3,4]. Anti-fungal agents, such as miconazole and clotrimazole, are also in use. Nevertheless, prolonged treatment is toxic to corneal cells and can cause side effects. In our previous studies, we found that commonly used multipurpose contact lens solutions are not effective against *Acanthamoeba* [5]. These findings are also supported by other authors [3,10,11,12]. Therefore, prevention is crucial to limit AK infections.

Nanoparticles (NPs) are currently studied in terms of their treatment and preventive capabilities against many microorganisms. Their attributes include small sizes, modifiable shapes, and various physiochemical properties. The mechanism of action of NPs is not yet fully understood. It includes a process of disruption of the cell membrane structure, the generation of reactive oxygen species (ROS) to interrupt respiratory chain enzymes inside the cell, the disturbance of the DNA replication process, and the inhibition of ATP-dependent (adenosine triphosphate-dependent) protein synthesis. The recognition of NPs by cells as a cell-surface receptor makes them an ideal drug carrier [13,14,15]. In recent years, silver nanoparticles (AgNPs) have been widely tested against a broad range of pathogens. The antibacterial activity of AgNPs has been described against *Escherichia coli*, *Pseudomonas aeruginosa*, *Staphylococcus aureus*, and *Bacillus subtilis* [16,17,18,19]. Furthermore, their anti-protozoal activity has been confirmed against *Toxoplasma gondii*, *Echinococcus granulosus*, *Schistosoma japonicum*, *Giardia intestinalis*, *Entamoeba histolytica*, *Leishmania* spp., and *Plasmodium* spp. [20,21,22,23,24,25,26]. The efficacy of AgNPs against *Acanthamoeba* spp. was described in our previous studies [14]. We showed that AgNPs possess anti-amoebic properties and the capability to reduce amoebae adhesion to the contact lens surface. The cytotoxic effect on human cells was limited [14]. Other authors showed that conjugations of AgNPs with various drugs and plant extracts might be promising strategies in AK treatment and prevention. Anwar et al. showed that conjugation of AgNPs with amphotericin B and nystatin increased the anti-amoebic capacity compared to the tested drugs alone [27]. The anti-amoebic properties of plant extracts have been also widely studied. Extracts of *Jatropha curcus*, *Jathopha gossypifolia*, and *Euphorbia milii* showed low human cell toxicity and high anti-amoebic activity when synthesized with AgNPs [28]. The anti-amoebic potential of tannic acid-modified silver nanoparticles (AgTANPs) was recently demonstrated by us [29]. Moreover, we also confirmed the anti-amoebic potential of AgNPs, AgTANPs, and gold nanoparticles (AuNPs) as contact lens solution components [5,30].

The aim of this study is to evaluate the anti-amoebic activity, cytotoxicity, and anti-adhesive properties of silver nanoparticles conjugated with five multipurpose contact lens solutions on the four Food and Drugs Administration (FDA) types of contact lenses against the *Acanthamoeba castellanii* NEFF strain.

## 2. Results

### 2.1. Activity Assay Results

**Figure 1:**
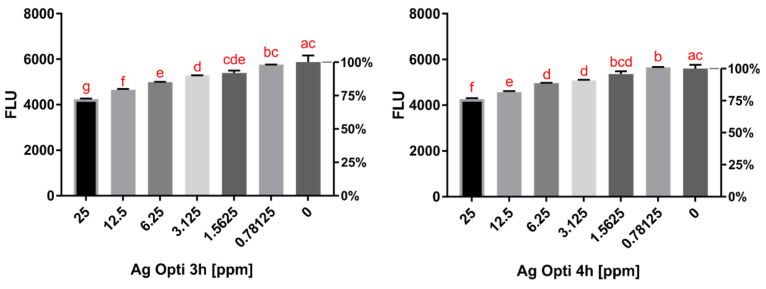
**Left:** Activity of *Acanthamoeba* trophozoites evaluated by fluorescence after **3 h** of incubation with Opti-Free (O-F) conjugated with AgNPs in different concentrations. **Right:** Activity of *Acanthamoeba* trophozoites evaluated by fluorescence after **4 h** of incubation with Opti-Free (O-F) conjugated with AgNPs in different concentrations. The same letters on different bars indicate no statistical significance (homogenous groups). “0” refers to the activity of the pure contact lens solution.

**Figure 2:**
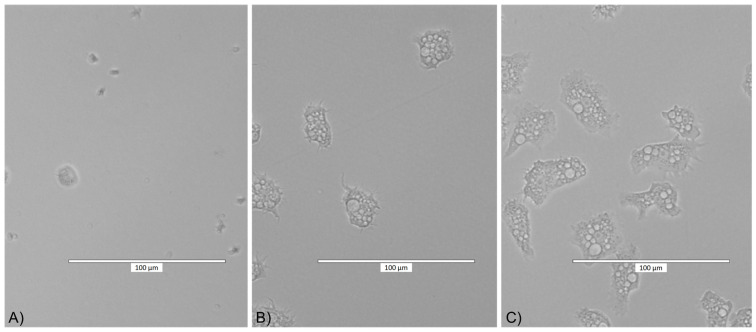
*Acanthamoeba* trophozoites after 4 h of incubation with (**A**) 25 ppm AgNPs conjugated with O-F, (**B**) O-F itself, and (**C**) peptone yeast glucose (PYG) medium.

**Figure 3:**
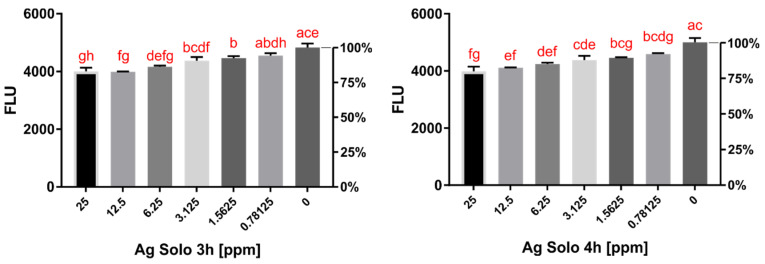
**Left:** Activity of *Acanthamoeba* trophozoites evaluated by fluorescence after **3 h** of incubation with Solo Care Aqua (SCA) conjugated with AgNPs in different concentrations. **Right:** Activity of *Acanthamoeba* trophozoites evaluated by fluorescence after **4 h** of incubation with Solo Care Aqua (SCA) conjugated with AgNPs in different concentrations. The same letters on different bars indicate no statistical significance (homogenous groups). “0” refers to the activity of the pure contact lens solution.

**Figure 4:**
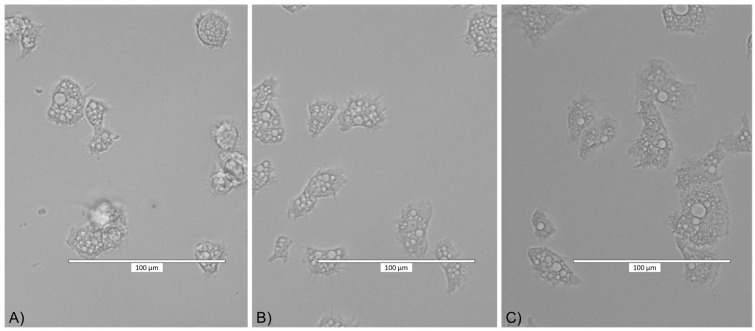
*Acanthamoeba* trophozoites after 4 h of incubation with (**A**) 25 ppm AgNPs conjugated with SCA, (**B**) SCA itself, and (**C**) PYG medium.

The activity of five contact lens solutions conjugated with AgNPs was tested using in vitro assays. Opti-Free (O-F) conjugated with AgNPs acted in a dose-dependent manner, reducing *Acanthamoeba* activity by up to 27.8% after 3 h and 23.8% after 4 h of incubation compared to the pure contact lens solution activity (Figure 1 and Figure 2). The increased activity was noted after up to 6 h of incubation, which is the minimum time of disinfection for O-F. SoloCare Aqua (SCA) conjugated with AgNPs reduced *Acanthamoeba* activity by up to 17.2% after 3 h and 20.3% after 4 h of incubation compared to the pure contact lens solution activity (Figure 3 and Figure 4). Relative efficiency was calculated to define the percentage increase of anti-amoebic activity. The three other multipurpose contact lens solutions, B-Lens (BL), Best View (BV), and Renu MultiPlus (ReNu), showed no statistically significant increase in anti-amoebic activity after up to 6 h of incubation.

### 2.2. Cytotoxicity Assay Results

**Figure 5:**
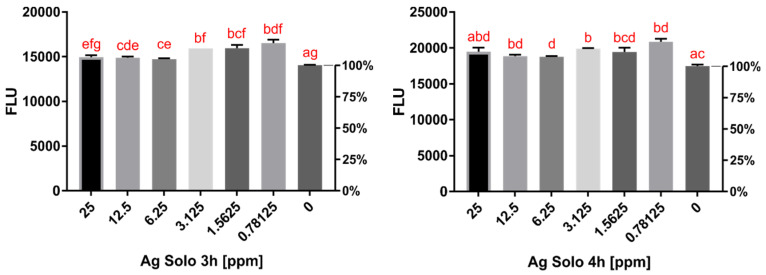
**Left:** Cytotoxicity on the macrophage cell line (J774A.1) evaluated by fluorescence after **3 h** of incubation with Solo Care Aqua (SCA) conjugated with AgNPs in different concentrations. **Right:** Cytotoxicity on the macrophage cell line (J774A.1) evaluated by fluorescence after **4 h** of incubation with Solo Care Aqua (SCA) conjugated with AgNPs in different concentrations. The same letters on different bars indicate no statistical significance (homogenous groups). “0” refers to the activity of the pure contact lens solution.

**Figure 6:**
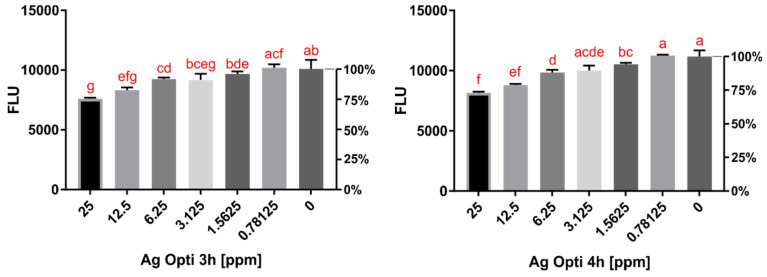
**Left:** Cytotoxicity on the macrophage cell line (J774A.1) evaluated by fluorescence after **3 h** of incubation with Opti-Free (O-F) conjugated with AgNPs in different concentrations. **Right:** Cytotoxicity on the macrophage cell line (J774A.1) evaluated by fluorescence after **4 h** of incubation with Opti-Free (O-F) conjugated with AgNPs in different concentrations. The same letters on different bars indicate no statistical significance (homogenous groups). “0” refers to the activity of the pure contact lens solution.

The cytotoxicity of O-F and SCA conjugated with AgNPs was tested using in vitro assays. SCA conjugated with AgNPs after 3 and 4 h surprisingly showed a reduction in the toxic effect against murine macrophages compared to the pure contact lens solution (Figure 5). O-F conjugated with AgNPs showed a dose-dependent increase in toxic effect compared to the pure contact lens solution (Figure 6).

### 2.3. Adhesion Assay Results

To verify and evaluate the influence of AgNPs conjugated with O-F and SCA on the adhesion properties of *Acanthamoeba* trophozoites, in vitro assays on four FDA groups of contact lenses were performed. The adhesion of trophozoites to the contact lens surface varied and depended on the contact lens FDA group. The strongest, i.e., monolayer adhesion, was observed in contact lens FDA groups 3 and 4. In FDA group 1, the adhesion was mild and irregular. FDA group 2 contact lenses were very delicate and broke during the experiments, preventing us from calculating the adhesion reduction (AR) correctly.

**Figure 7:**
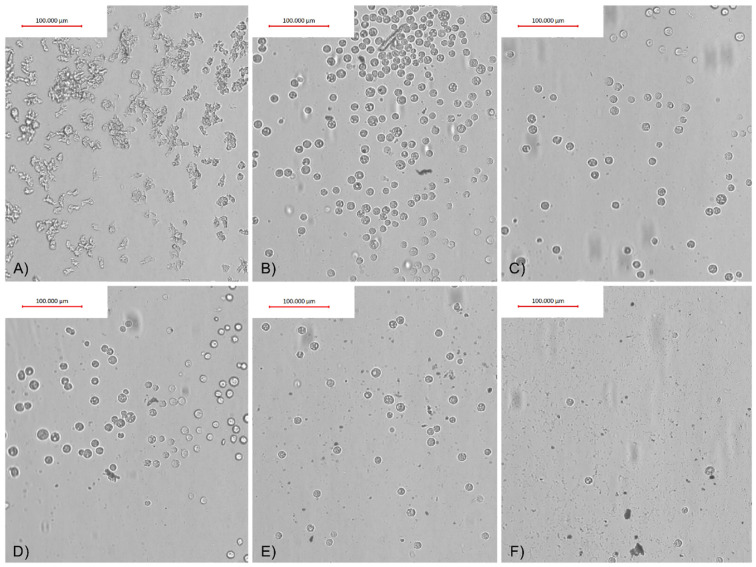
*Acnthamoeba castellanii* visualized results of the adhesion reduction (AR) to FDA group 4 contact lenses’ surface after treatment with O-F conjugated with AgNPs. (**A**) Water control; (**B**) O-F control; (**C**) AgNPs at 3.125 ppm; (**D**) AgNPs at 6.25 ppm; (**E**) AgNPs at 12.5 ppm; (**F**) AgNPs at 25 ppm.

**Figure 8:**
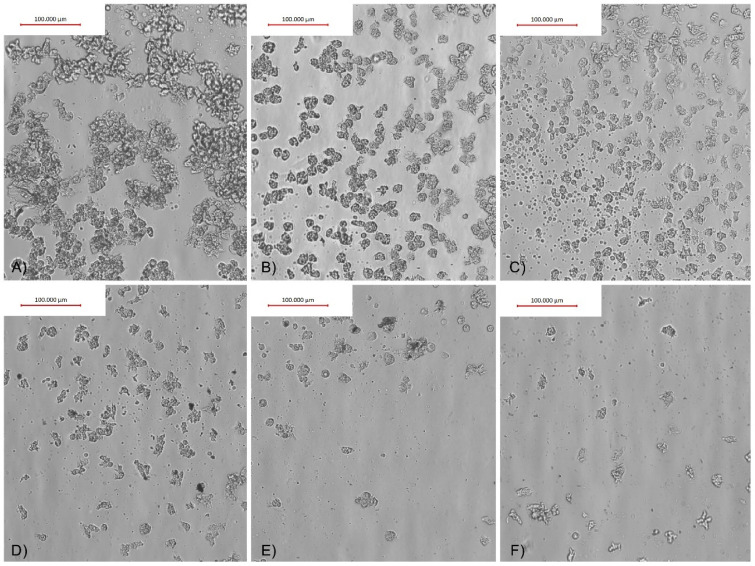
*Acnthamoeba castellanii* visualized results of the adhesion reduction (AR) to FDA group 1 contact lenses’ surface after treatment with SCA conjugated with AgNPs. (**A**) Water control; (**B**) O-F control; (**C**) AgNPs at 3.125 ppm; (**D**) AgNPs at 6.25 ppm; (**E**) AgNPs at 12.5 ppm; (**F**) AgNPs at 25 ppm.

The adhesion reduction (AR) was dependent on the AgNPs and observed in FDA groups 1, 3, and 4 contact lenses treated with O-F + AgNPs, and FDA groups 1 and 4 treated with SCA + AgNPs (Table 1). The best adhesion reduction (AR) is visualized in Figure 7 and Figure 8.

## 3. Discussion

Silver nanoparticles (AgNPs) have been widely tested and found to be effective against viruses and bacteria [15]. Recently, we proposed silver nanoparticles as a novel approach in *Acanthamoeba* keratitis therapy and prevention. We also tested AgNPs and tannic acid-modified AgNPs against *A. castellanii* [14,29]. In both cases, significant anti-amoebic activity, combined with low cytotoxicity to human fibroblasts, was confirmed. Nanoparticles were well absorbed by the trophozoites and did not induce the encystation process [14,29]. Similar studies have been performed by other authors and confirmed that nanoparticles could be effective against *Acanthamoeba* trophozoites and cysts [13,27,31,32].

Strengthening the anti-amoebic properties of contact lens solutions is an urgent need. One way to achieve this is to conjugate them with other anti-amoebic agents. We previously conjugated contact lens solutions with tannic acid-modified AgNPs and obtained satisfying results in reducing the activity of amoebae [30]. In the current study, we tested pure AgNPs conjugated with five contact lens solutions. We observed increased anti-amoebic activity in two contact lens solution conjugates. Moreover, conjugation of AgNPs with SCA did not increase cytotoxicity in murine macrophages, which is very promising in terms of the possible practical use of this conjugation. Studies performed by other authors also proved that NPs conjugated with other agents can be a promising method of *Acanthamoeba* eradication. Anwar et al. presented satisfying results on the anti-*Acanthamoeba* activity of AgNPs conjugated with metformin and guanabenz. Additionally, the metformin-coated AgNPs limited encystation and inhibited the excystation of the amoebae [33,34]. Similar results were obtained for AgNPs and tetrazole conjugates [35]. Anti-fungal drugs such as nystatin, fluconazole, and amphotericin B conjugated with AgNPs also showed increased anti-amoebic activity [27].

*Acanthamoeba* adhesion to the contact lens and transmission from there to the corneal epithelium surface is the first step in AK development [7]. In our previous studies, we tested the adherence of the amoebae to four FDA types of contact lenses [14]. The obtained data showed significant differences in adhesion rate between ionized and non-ionized contact lens material. The adhesion to lenses composed of ionized material (FDA groups 3 and 4) was much higher and stronger compared to non-ionized lenses (FDA groups 1 and 2). The highest adhesion was noticed in FDA group 4 contact lenses containing Methafilcon A with high hydration (55%) and no silicone content [14]. Similar results were obtained in the current study, where the strongest adhesion was observed in contact lenses belonging to FDA groups 3 and 4. The results obtained by us suggest that ionization of the contact lens material is the main factor favoring *Acanthamoeba* adhesion. These conclusions are supported by findings revealed by other authors. Research conducted by Lee et al. showed that contact lenses made from Etafilcon A (high water content and ionic material) showed greater *Acanthamoeba* adhesion to their surface [36]. However, Omaña-Molina et al. performed studies on contact lenses containing Lotrafilcon A, Galyfilcon A, and Comfilcon A, and revealed that amoebae showed better adhesion to the Lotrafilcon A non-ionic material, which contains the lowest amount of water (33%) [37]. On the other hand, Bakey et al. found no significant difference in *Acanthamoeba* adhesion between contact lenses with high and low water contents [38].

The action of the contact lens solutions on amoebae adhesion to the contact lens surface was tested by us. The findings revealed that contact lens solutions showed various anti-adhesion effects that depend on the FDA group of the contact lenses tested. Particularly, O-F solution containing TearGlyde did not reduce *Acanthamoeba* adhesion in FDA groups 3 and 4 contact lenses. SCA containing Polyhexanide 0.0001% reduced the adhesion of *Acanthamoeba* in FDA group 4 contact lenses by 68%. In contrast, Lee et al. showed that contact lens solutions containing myristamidopropyl dimethylamine effectively decreased the number of trophozoites adhered to the contact lens surface [39]. Logically, decreasing *Acanthamoeba* adhesion to the contact lens surface should be the first step in preventing AK infection in the contact lens user population. In this regard, we have successfully tested contact lens solutions conjugated with silver nanoparticles (AgNPs) and achieved a significant reduction in *Acanthamoeba* adhesion without increased cytotoxicity [14]. The significant results in amoebae adhesion reduction were obtained starting from 3.125 ppm concentration of AgNPs, however, the best cytotoxicity/anti-adhesive ratio was revealed in conjugation with SCA at a concentration of 12.5–25 ppm. Similar results were obtained for anti-amoebic activities of tested nanoconjugates and are in accordance with our previous studies published in 2019 [5]. It is also noteworthy that similar effectiveness in the anti-adhesive activity of nanoconjugates might be achieved with up to ten times lower concentrations of nanoparticles compared to pure AgNPs, at 6.25 ppm for AgNPs+O-F and 12.5 ppm for AgNPs+SCA compared to 60 ppm for pure AgNPs. However, the effective anti-amoebic activity of nanoconjugates and pure AgNPs has been achieved at the same concentration of nanoparticles [14]. The obtained results suggest (see Figure 7 and Figure 8) that the anti-adhesive mode of action of nanoconjugates does not change the survival status of the amoebae but rather affects the cell shape by reducing the number of acanthopodia that facilitate the adhesion process [36]. Seung-Mok et al. applied multipurpose contact lens solutions combined with the autophagy inhibitors 3-methyladenine and chloroquine. Their results revealed a significant reduction in adhered amoebae compared to pure contact lens solutions [40]. Mitsuwan et al. applied *Curcuma longa* extract and curcumin to contact lenses covered with *Acanthamoeba*. Their results also revealed significant inhibition of *Acanthamoeba triangularis* adhesion to the contact lens surface [41]. Altogether, the obtained results suggest great potential for improving the efficacy of contact lens solutions through conjugation with other agents and nanoparticles.

## 4. Conclusions

The anti-amoebic activity, measured within the minimum time of disinfection recommended by the manufacturer of the multipurpose contact lens solutions, is limited. The ionization of the contact lens material is one of the factors influencing the adhesion of *Acanthamoeba* to the contact lens surface. Contact lens solutions do not protect the contact lens surface from *Acanthamoeba* trophozoite adhesion. The anti-adhesive properties of contact lens solutions might be reinforced, without increased cytotoxic effect, if conjugated with AgNPs. In conclusion, AgNPs should be considered as a contact lens solution component that may reduce the risk of *Acanthamoeba* keratitis cases among contact lens users. Further studies are needed to evaluate the influence of the nanoconjugates on the *Acanthamoeba* cyst stage.

## 5. Materials and Methods

### 5.1. Acanthamoeba and Macrophage Cultivation

For the activity assays, *Acanthamoeba castellanii* NEFF strain (ATCC 30010; LG Promochem, Barcelona, Spain) from the American Type Culture Collection was used. The strain was cultured axenically in culture tissue flasks at 27 °C. Then, in a peptone yeast glucose (PYG) medium (0.75% (*w*/*v*) containing protease peptone, 0.75% (*w*/*v*) yeast extract, and 1.5% (*w*/*v*) glucose with 10 mg of gentamicin mL^−1^ (Biochrom AG, Cultek, Granollers, Barcelona, Spain). The tests were performed at the Institute of Tropical Diseases and Public Health, University of La Laguna, Spain, and at the Department of Medical Biology, Medical University of Warsaw, Poland. The strains were subcultured for three days before the assays and observed under a Leica DMIL inverted microscope (Leica, Wetzlar, Germany).

For the toxicity assays, the murine macrophage J774A.1 (ATCC TIB-67) cell line was used. The cell line was cultured in Dulbecco’s Modified Eagle´s medium (DMEM, *w*/*v*) supplemented with 10% (*v*/*v*) fetal bovine serum with 10 µg/mL gentamicin (Sigma-Aldrich, Madrid, Spain). The conditions of culture were 37 °C and a 5% CO_2_ atmosphere.

For the experiments performed in this study, the *Acanthamoeba* and macrophage strains were used in their logarithmic phase of growth.

### 5.2. Nanoparticles

The original stock solutions of AgNPs in concentrations of 1000 ppm were diluted in miliQ water to obtain a 250 ppm sample concentration. The storage conditions were 27 °C in darkness until the experiments. The stock solutions were sonicated before performing the assays. The AgNPs used in this study were kindly provided by the Department of Nanobiotechnology and Experimental Ecology, Institute of Biology, Warsaw University of Life Sciences, Poland.

AgNPs were acquired from Sigma Aldrich, Poole, UK (Cat. No. 576832). By transmission electron microscopy (TEM) using a JEOL JEM-1220 TE microscope at 80 KeV (JEOL Ltd., Tokyo, Japan), with a Morada 11-megapixel camera (Olympus Corporation, Tokyo, Japan), the size and distribution of the AgNPs were defined. Their size was between 15 to 30 nm (average value: 22.3 ± 12.41 nm), as previously mentioned [14].

The Zeta potential of the AgNPs was measured by the electrophoretic light-scattering method using a Zetasizer Nano-ZS90 (Malvern, Worcestershire, UK). The sample was measured after 120 s of stabilization, in 20 replicates. The stabilization conditions were at 25 °C and pH 8.6.

### 5.3. Contact Lens Solutions

Five multipurpose solutions were tested: SCA, O-F, ReNu, BL, and BV. Those solutions represent five of the most popular types of solutions used for contact lens disinfection in Poland. The ingredients of the tested contact lens solutions are presented below, in Table 2. All multipurpose solutions used in this study were acquired from authorized agents.

### 5.4. Activity Assays

AgNPs at concentrations of 25, 12.5, 6.25, 3.125, 1.5625, and 0.78125 ppm were conjugated with five contact lens solutions and tested for their anti-amoebic activity.

The activity of the selected NPs against the trophozoite stage of the *Acanthamoeba castellanii* NEFF strain was specified during the in vitro experiments using a colorimetric assay, based on the oxide-reduction of the alamarBlue^®^ reagent (Life Technologies, Barcelona, Spain), as previously described.

The cell suspension necessary for the experiments was 2.5 × 10^4^ cells/well. To achieve this concentration, the trophozoites were counted by a Countess II FL automatic cell counter (Thermo Fisher Scientific, Madrid, Spain). 50 µL of the suspension was seeded in each well of a 96-well plate (Thermo Fisher Scientific, Madrid, Spain). After half an hour, the amoebae cells were adhering to the bottom of the wells.

During this period, the AgNPs were diluted in miliQ water in a deep well plate. After that, 40 μL of each contact lens solution and 10 μL of the AgNPs’ serial dilution were added into the plate wells. *Acanthamoeba* trophozoites were also incubated with only 40 µL of contact lens solution and 10 µL of miliQ water despite the NPs, as a negative control. The positive control constituted *Acanthamoeba* trophozoites incubated with only 50 µL of PYG medium. Finally, 10 μL of alamarBlue^®^ reagent was seeded into all of the wells. The plate incubation conditions were 28 °C with slight agitation. Next, the emitted fluorescence was measured over periods of 0, 15, and 30 s, and 1, 2, 3, 4, 5, and 6 h with an EnSpire^®^ Multimode Plate Reader (Perkin Elmer, Madrid, Spain). The recommended wavelengths are 570 nm and 630 nm.

To calculate the percentage of growth inhibition, fluorescence bar graphs obtained from GraphPad software were generated. All experiments were performed in duplicate. The statistical analysis was obtained using a one-way ANOVA in GraphPad. The mean values and standard deviations were calculated. The *p* value was designated at the level <0.05 as statistically significant. *Acanthamoeba* in all the wells was observed using an Evos fl Cell Imaging System.

### 5.5. Cytotoxicity Assays

To investigate and compare the cytotoxicity of the AgNPs conjugated with the five selected contact lens solutions, an assay with murine macrophage cell line J774A.1 (ATCC TIB-67) was performed. The medium suitable for a macrophage cell line is RPMI (Roswell Park Memorial Institute) 1640 medium without phenol red (Roswell Park Memorial Institute, Thermo Fisher Scientific Inc., Waltham, MA, USA). To achieve the concentration of 10^5^ cells per each well the macrophages were counted by a Countess II FL automatic cell counter (Thermo Fisher Scientific, Madrid, Spain) and 50 µL were seeded into each well of 96-well plates. Then, 40 µL of each contact lens solution was added to each well along with serial dilutions of the NPs (10 µL) to achieve a final volume of 100 µL per well, as described above. Macrophages were also incubated with only 40 µL of contact lens solution and 10 µL of miliQ water, instead of the NPs, as a negative control. The positive control constituted macrophages incubated with only 50 µL of RPMI medium. In the end, 10 µL of alamarBlue^®^ reagent was added into each well. The plate incubation conditions were 37 °C and a 5% CO_2_ atmosphere. The emitted fluorescence was measured over periods of 0, 15, and 30 s, and 1, 2, 3, 4, 5, and 6 h with an EnSpire^®^ Multimode Plate Reader (Perkin Elmer, Madrid, Spain). The recommended wavelengths are 570 and 630 nm.

To calculate the percentage of cytotoxicity, the statistical analysis software GraphPad was used as previously described. All experiments were performed in duplicate. Macrophages in both the controls and the tested assays were observed by an Evos fl Cell Imaging System.

### 5.6. Adhesion Assays

To investigate the action of AgNPs conjugated with the two selected multipurpose contact lens solutions (SCA and O-F) on the *Acanthamoeba* NEFF strain’s adhesion skills, assays using four types of hydrogel contact lenses (Table 3) classified by the FDA were performed. To perform the experiments, the contact lenses were placed into a 24-well microtitrate plate and exposed to 1000 µL of the *Acanthamoeba* suspension at a concentration of 10^5^ cells per contact lens. After 90 min, at room temperature, *Acanthamoeba* cells were adhering to the contact lens surface. After this time, each contact lens coated with the *Acanthamoeba* was transferred to another well with saline solution. The number of attached cells was monitored using an inverted microscope OPTA-TECH MW50 with an OPTA-TECH MI5FL 5 MP digital camera. Next, the coated contact lenses were exposed to 400 µL of each contact lens solution and 100 µL of AgNPs at concentrations of 25, 12.5, 6.25, and 3.125 ppm. As controls, the contact lenses were incubated with only 400 µL of each contact lens solution and 100 µL of miliQ water, instead of NPs. The incubation periods were 4 and 6 h at room temperature, depending on the contact lens solution used. Subsequently, the contact lenses were transferred to fresh wells with saline solution, and the number of attached *Acanthamoeba* cells was counted. The experiments were performed in triplicate and the cells were observed by an inverted microscope OPTA-TECH MW50 with an OPTA-TECH MI5FL 5 MP digital camera. *Acanthamoeba* adhesion reduction (AR) was calculated using the formula presented below:

AR = (nc − nt)/nc × 100%

where nc is the number of attached amoebae in the control well and nt is the number of attached amoebae in the test well.

## Figures and Tables

**Table 1:** *Acanthamoeba castellanii* results of the adhesion reduction (AR) to the contact lens surface.

	FDA 1	FDA 2	FDA 3	FDA 4
SCA	8.8 ± 4.2	no activity	no activity	63.5 ± 14.0
SCA + 25 ppm AgNPs	90.0 ± 2.2	no activity	51.6 ± 9.1	95.2 ± 1.5
SCA + 12.5 ppm AgNPs	74.4 ± 0.8	no activity	50.9 ± 6.2	93.1 ± 2.3
SCA + 6.25 ppm AgNPs	36.9 ± 0.4	no activity	21.1 ± 20.5	89.0 ± 2.4
SCA + 3.125 ppm AgNPs	unreliable data	no activity	37.9 ± 5.0	80.3 ± 6.1
O-F	13.2 ± 23.2	unreliable data	no activity	no activity
O-F + 25 ppm AgNPs	94.7 ± 3.7	unreliable data	95.3 ± 0.5	94.7 ± 0.7
O-F + 12.5 ppm AgNPs	91.9 ± 2.1	unreliable data	69.8 ± 2.9	82.2 ± 0.3
O-F + 6.25 ppm AgNPs	76.7 ± 3.3	unreliable data	55.6 ± 13.5	62.7 ± 0.7
O-F + 3.125 ppm AgNPs	56.4 ± 0.4	unreliable data	6.1 ± 55.8	55.7 ± 19.0

**Table 2:** Ingredients of the multipurpose contact lens solutions and minimum disinfection times recommended by the manufacturers.

Manufacturer	Solution	Ingredients	Minimum Disinfection Time (h)
Menicon	Solo Care Aqua (SCA)	Polyhexanide 0.0001%, Hydrolock (dexpanthenol, sorbitol), sodium phosphate, tromethamine, poloxamer 407, disodium edetate	4
Alcon	Opti Free (O-F)	TearGlyde (Tetronic 1304, nonanoyl ethylenediaminetriacetic acid), Polyquad (polyquaternium-1) 0.001%, Aldox (myristamidopropyl dimethylamine) 0.0005%	6
Bausch + Lomb	ReNu Multiplus (ReNu)	Hydranate (hydroxyalkylphosphonate) 0.03%, boric acid, edetate disodium, poloxamine 1%, sodium borate, sodium chloride, preserved with Dymed (polyaminopropyl biguanide 0.0001%)	4
B-Lens	B-Lens (BL)	Polyheksametylene biguanide (PHMB) 0.0001%, Pluronic F 1%, Panthenol 1.5%.	4
Best View	Best View (BV)	PHMB 0.0001%, Ethylenediaminetetraacetic acid (EDTA), 0.01%, Pluronic	6

**Table 3:** Characterization of the FDA types of the hydrogel contact lenses used in the study.

Polymer	FDA Group	Water Content	Ionic	Silicon Content	Manufacturer
Senofilcon A	1	38%	no	yes	ACUVUE oasys
Nelfilcon A	2	69%	no	no	Dailies
Balafilcon A	3	36%	yes	yes	Baush + Lomb PureVision
Methafilcon A	4	55%	yes	no	FitView

## Data Availability

The datasets used and/or analyzed during the current study are available from the corresponding author on reasonable request.
